# Efficacy of Intrauterine Perfusion of Cyclosporin A for Intractable Recurrent Spontaneous Abortion Patients With Endometrial Alloimmune Disorders: A Randomized Controlled Trial

**DOI:** 10.3389/fphys.2021.737878

**Published:** 2021-09-06

**Authors:** Long Zhao, Lijuan Qi, Jinhua Fu, Shuqin Bi, Lin Li, Yinghui Fu

**Affiliations:** ^1^Department of Nephrology, The Affiliated Hospital of Qingdao University, Qingdao, China; ^2^Department of Obstetrics, Qingdao Jinhua Gynecology Hospital, Qingdao, China

**Keywords:** intrauterine perfusion of CsA, recurrent spontaneous abortion, uterine natural killer cells, CD56, CD57

## Abstract

**Objective:**

To explore the therapeutic efficacy of intrauterine perfusion of cyclosporin A (CsA) in intractable recurrent spontaneous abortion (RSA) patients with endometrial alloimmune dysfunction.

**Methods:**

This is a randomized controlled trial (RCT) of patients with intractable RSA with endometrial alloimmune disorders. A total of 201 women were enrolled, all of whom had at least 3 serial abortions and endometrial alloimmune dysfunction. Participants were randomly assigned to two groups. The CsA group (*n* = 101) received intrauterine infusion of 250 mg CsA on the 3rd and 7th days after menstruation for 2 menstrual cycles, while the placebo group (*n* = 100) received placebo. The birth of healthy, deformity-free babies was the main study outcome.

**Results:**

In total, 75 (74.26%) women in the CsA group and 59 (59.00%) women in the placebo group gave birth to healthy babies [*P* = 0.01, *OR* = 2.01; 95% *CI* (1.10∼3.65)]. Compared to the placebo group, the CsA group had dramatically lower endometrial CD56^+^ cell and CD57^+^ cell concentrations at the luteal phase of the second menstrual cycle (*P* < 0.05).

**Conclusion:**

Intrauterine perfusion of CsA was confirmed to be a promising approach for the treatment of intractable alloimmune RSA.

## Introduction

The occurrence of two or more consecutive spontaneous abortions is defined as recurrent spontaneous abortion (RSA) (Fu, 2015). RSA morbidity is estimated to affect 5% of reproductive-aged females (Fu, 2015). Currently recognized causes include infectious diseases, chromosomal abnormalities, uterine abnormalities, endocrine factors, autoimmune diseases, alloimmune disorders, and endometrial microenvironment disorders, among others (Shahine and Lathi, 2014; Fu et al., 2015). Among them, endometrial alloimmune disorders might be the core problem (Craciunas et al., 2019). During the embeddedness phase, the endometrium sustains further embeddedness induced by chemokines and adhesion molecules, growth factors, cytokines and immune cells (van Mourik et al., 2009). Additionally, uterine natural killer (uNK) cells, which are involved in alloimmunity, account for 65–70% of lymphocytes in the endometrium (Jiang et al., 2017). Since 2018, our center has used low-molecular-weight heparin, aspirin, prednisone, lymphocyte immunotherapy for the husband and intravenous immunoglobulin (IVIG) to treat RSA patients. The success ratio is approximately 84%, yet the failure rate is still 16%. The key reason for this is that through conventional treatment, endometrial microenvironmental disorders are still not improved, so there continues to be no effective treatment.

Uterine natural killer (uNK) cells proliferate after ovulation, and by the late secretion stage, they make up over 30% of immune cells. In early pregnancy, CD56^+^ cells remain upregulated, accounting for 70% of immune cells at the maternal-fetal interface. UNK cells may play a key role in trophoblast invasion and migration and in the placenta. The endometrial uNK cell concentration in women with RSA is markedly changed, but conclusions remain debatable (Quenby et al., 1999; Matteo et al., 2007; Tuckerman et al., 2007, 2010; Seshadri and Sunkara, 2014).

CD57 is often considered to be a biomarker for the final differentiation of cytotoxic T cells, while Lopez-Vergès S recently reported that CD57 positivity might indicate a subtype of NK cells that are more mature (Lopez-Vergès et al., 2010). CD57^+^ NK cells were observed in both peripheral blood and the endometrium, yet significant tendencies were not detected in either the absolute number or concentration (Laird et al., 2003; Russell et al., 2011; Dorwal et al., 2015). Although the uNK cells of the endometrium were considered to have enhanced cytokine production and low cytolytic toxicity, a high number of CD57^+^ NK cells were detected in the deciduae of RSA patients. Additionally, local cytokines were able to activate those cells to damage trophoblast layers (Vassiliadou and Bulmer, 1996). However, the function and distribution of endometrial CD57^+^ NK cells in female RSA remain unclear.

Cyclosporin A (CsA), which has become a first-line treatment drug for kidney transplantation combined with pregnancy, can significantly inhibit the autoimmune response and is widely used in patients after organ transplantation (Perales-Puchalt et al., 2012). Conventional doses of prednisone and CsA have been taken for a long time to prevent rejection of the transplant in pregnant patients after organ transplantation, and studies have shown that there are no adverse effects on fetuses and newborns caused by the drugs (Perales-Puchalt et al., 2012). CsA may play a dual role in maternal-fetal immune regulation, which can not only inhibit maternal immunological rejection of the embryonic antigen but can also promote the growth of trophoblast cells (Zhou et al., 2008; Azizi et al., 2019). Our previous research has also shown that oral CsA can inhibit NK cells and increase the live birth ratio of patients with intractable immune RSA, which indicates that intrauterine perfusion of CsA may be an attractive therapeutic strategy for RSA (Porter et al., 2006; Fu et al., 2016).

Intrauterine perfusion is a kind of local intrauterine drug administration therapy wherein a transplanted tube is inserted through the vagina into the cervical opening so that liquid can be slowly pushed into the uterine cavity. In contrast to intravenous injection, intrauterine perfusion does not administer therapy directly into the blood circulation but instead relies mainly on absorption of the agent through the mucosal system. Intrauterine perfusion of G-CSF, HCG, peripheral blood mononuclear cells (PBMCs) and other drugs is currently widely used in the field of female reproduction to improve endometrial receptivity and embryo implantation rates. However, intrauterine perfusion of CsA in RSA patients has not yet been reported (Nazari et al., 2016).

Here, we performed intrauterine perfusion of CsA in consenting patients for whom conventional therapy (low-molecular-weight heparin, aspirin, prednisone, lymphocyte immunotherapy for the husband, and IVIG) had failed.

## Materials and Methods

Patients with intractable RSA (no previous successful pregnancies) admitted to Qingdao Jinhua Hospital between August 2018 and December 2020 were enrolled. The definition and diagnostic basis of all pregnancy events were in line with the ESHRE Early Pregnancy Special Interest Group. The Medical Ethics Committee of Qingdao Jinhua Hospital reviewed and authorized the present research. This randomized controlled trial (RCT) was administered in accordance with the “Helsinki Declaration on Medical Research Involving the Human Body” and the laws of China.

The inclusion criteria were as follows: women aged less than 40 years, with more than 3 prior miscarriages, with a CD56^+^ cell concentration >5% and CD57^+^ concentration >1% among endometrial cells in the luteal phase (Lash et al., 2016), and with conventional treatment failure (low-molecular-weight heparin, aspirin, prednisone, lymphocyte immunotherapy for the husband, IVIG). Patients were negative for other causes of RSA including uterine defects, an abnormal karyotype, endocrine problems, infections, autoimmune deficiency, thrombosis or coagulation defects. All participants were tested multiple times, and only couples without malformations or chromosome abnormality were included. No abnormalities were detected in the semen analysis of male partners, and no uterine abnormalities were detected by uteroscopy and/or pelvic ultrasound. Thyroid tests (anti-peroxidase, antioxidant globulin antibodies, TSH, free T3 and free T4), hormone blood tests (dehydroepiandrosterone, FSH, testosterone, LH, progesterone, prolactin, estrone, 17β-estradiol, D4 androstenedione, 17αOH progesterone), insulin release tests and oral glucose tolerance tests (OGTTs) were all within the normal range. In the Coombs test, anti-phospholipid autoantibodies (anticoagulants for lupus, anti-cardiolipin antibodies) and other autoantibodies (anti-gastric parietal cells, anti-mitochondria, anti-ENA, and anti-nuclear) were normal. Blood tests for Toxoplasma, rubella, herpesvirus types I and II, cytomegalovirus, mycoplasma and chlamydia were negative. Antithrombin III, thrombin C, thrombin S, homocysteine and thrombin factors XII, VIII, VII, V, and II were not abnormal. Moreover, participants had normal embryo-tissue karyotypes during the last abortion (during conventional treatment), which were 46/XY for 105 abortions and 46/XX for 96 abortions. All the participants had fertile partners who had no fertility barriers, and no one had come through *in vitro* fertilization-embryo transfer (IVF-ET).

A total of 209 women were deemed qualified for this study, and 201 consented to participate. In addition to the lack of information on the developmental toxicity of intrauterine CsA perfusion, all participants were informed that there might be some risks to children or parents. A random number sequence generated by a computer was used to randomize the participants. They were randomly assigned to either the primary treatment (low-molecular-weight heparin, aspirin, prednisone, lymphocyte immunotherapy for the husband, IVIG) plus intrauterine perfusion of CsA or the primary treatment plus placebo. The patients did not know their group assignments. Participants could not re-enter the study and could be randomized only once. A total of 101 women in the CsA group received intrauterine perfusion of CsA (Sandimmun, Novartis Pharma Schweiz AG, Switzerland) at a dose of 250 mg on the 3rd and 7th days after menstruation for 2 menstrual cycles, in which contraception was carried out. Endometrial biopsies were performed at the luteal phase of the second menstrual cycle.

One hundred patients in the placebo group were given intrauterine perfusion of normal saline at the same dose and at the same time. Endometrial CD56^+^ cell and CD57^+^ cell concentrations were measured in the second luteal phase in both groups. In this study, all patients became pregnant within 3 months after 2 months of treatment. Every participant was followed up at Qingdao Jinhua Hospital throughout gestation, with transvaginal ultrasonography every 2 weeks from 4 to 12 weeks of gestation to observe embryo growth and the heartbeat or to confirm abortion (on the basis of the criteria of ESHRE). All newborns were examined by pediatricians to rule out malformations. Ultrasonography was used to measure the gestational age at abortion by means of the fetal sac size and crown-rump length. Birth of healthy babies without malformations was the main outcome. The secondary outcomes were side effects of treatments, neonatal weight and probable gestational complications (preeclampsia, preterm delivery, bleeding and thrombosis, gestational hypertension, gestational diabetes mellitus).

Endometrial samples were collected using an endometrial curette. The tissue was cut into small pieces with ophthalmic scissors on a 200-mesh stainless steel nylon net. The rinsed suspension was collected into a centrifuge tube until the tissue blocks were ground (depending on the situation). Then, the concentrations of CD56^+^ cells and CD57^+^ cells were measured by flow cytometry (BeamCyte-1026, Beam Diag, China).

SPSS version 21.0 (SPSS Inc., United States) was applied to carry out statistical analysis. When applicable to continuous variables, the Bonferroni correction was performed for multiple comparisons, and the two-tailed Student’s *t*-test was performed for unpaired data. When applicable to discontinuous variables, the *χ*^2^ and Fishers exac*t*-test were applied. *P* < 0.05 was considered statistically significant.

## Results

### Basic Information of the Patients

There was no difference in body mass index (BMI), number of previous miscarriages or age at the beginning of pregnancy or gestational age of miscarriage between the two groups (Table 1). No participants dropped out of the study. Participants were completely followed up through gestation. No violations of the research protocol were observed.

**TABLE 1:** The basic information of the patients.

	Number of patients	Age (years)	BMI	Smokers*	Number of previous miscarriages	Gestational week at miscarriage
CsA	101	34.7 ± 2.6	27.9 ± 2.0	1	5.3 ± 0.4	6.4 ± 1.1
Placebo	100	35.0 ± 2.9	27.4 ± 1.8	0	5.4 ± 0.3	6.3 ± 1.2

*More than 10 cigarettes per day.

## The Main Outcome and Secondary Outcomes

There were 75 live births (74.26%) among 101 patients in the CsA group and 59 live births (59.00%) among 100 patients with placebo, and the difference was statistically significant [*P* = 0.01, odds ratio (OR) = 2.01; 95% confidence interval (CI) (1.10–3.65)]. *The number of patients who needed treatment* (NNT) per added live birth was 6.55. As shown in Table 2, pregnancy complications in the CsA group were decreased compared to those in the placebo group. There were no differences in treatment side effects, neonatal weight or gestational age between the two groups. In the CsA group, 2 patients had a skin rash, and 1 patient developed gestational hypertension; in the placebo group, 1 patient developed a skin rash, and 8 patients developed gestational hypertension. One newborn in the CsA group had a malformation (aberrant subclavian artery), which required no treatment as assessed by a cardiovascular physician. One newborn in the control group had a malformation (ventricular septal defect), which can possibly be cured by interventional therapy. Among the 65 abortions, there were karyotypes from 45 fetal tissues, and 11 were abnormal karyotypes, with 6 in the CsA group and 5 in the control group. Of these 34 normal karyotypes, 16 cases were 46XX type, and 18 cases were 46XY type.

**TABLE 2:** The main outcome and secondary outcomes.

	Number of live births (%)	Number of miscarriages (%)	Gestational week at miscarriage (mean ± SD)	Newborn Apgar score		Newborn weight (g, mean ± SD)	Side effects		
						
				1 min	5 min		Skin rash	Gestational hypertension	Congenital malformation
Intrauterine perfusion of CsA	75 (74.26)	24 (25.74)	7.1 ± 1.3	9.7 ± 0.6	9.9 ± 0.3	3,070 ± 232	2	1	0
Placebo	59 (59.00)	41 (41.00)	7.0 ± 1.0	9.6 ± 0.5	9.9 ± 0.4	3,110 ± 225	1	8	1
*P*-value	<0.01	<0.01	>0.05	>0.05	>0.05	>0.05	>0.05	<0.05	>0.05

## Comparison of Endometrial CD56^+^ Cell and CD57^+^ Cell Concentrations

**FIGURE 1:**
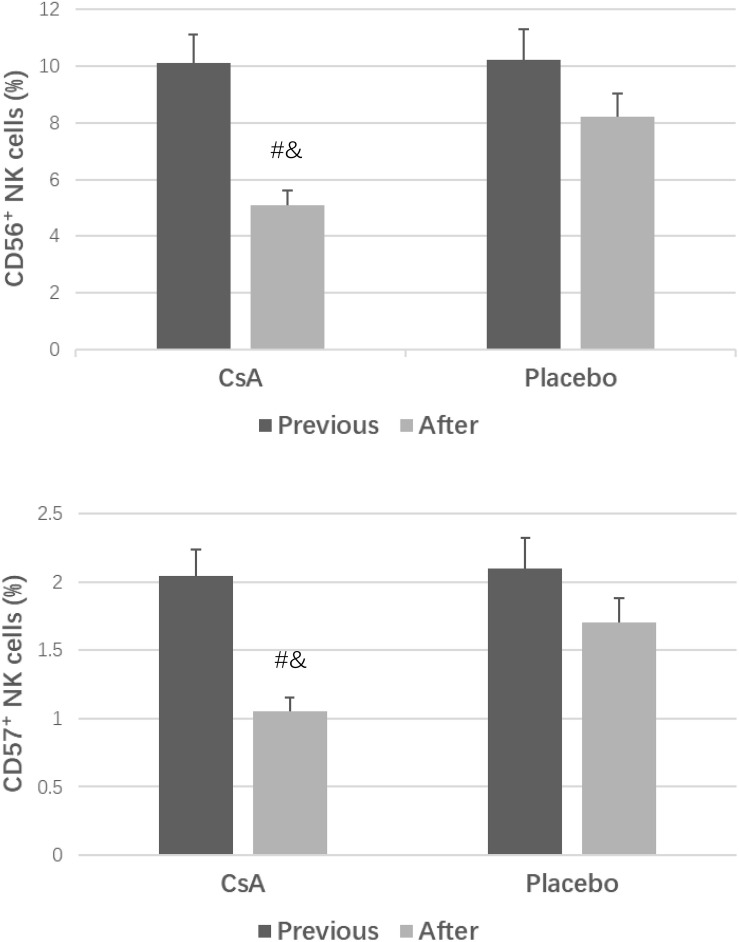
CD56^+^ cell and CD57^+^ cell concentrations in the two groups. ^#^*P* < 0.05 versus the placebo group, ^&^*P* < 0.05 versus previous treatment.

As shown in Figure 1, compared to those in the placebo group, the endometrial CD56^+^ NK cell and CD57^+^ NK cell concentrations in the CsA group were decreased at the second luteal phase (*P* < 0.05). Moreover, after intrauterine perfusion of CsA, the endometrial CD56^+^ cell and CD57^+^ cell concentrations were dramatically decreased.

## Discussion

Research results for RSA treatment have differed to varying degrees for three reasons. First, all research tends to favor spontaneous problem solving. Second, the sample is limited. Third, the inclusion criteria (in particular, previous miscarriages and patient age) of those studies varied widely, while the abortion risk increased with previous miscarriages and maternal age.

In the present study, intrauterine perfusion of CsA significantly affected intractable alloimmune RSA, significantly increased the success rate and reduced the abortion rate. Before this, only one retrospective cohort study had shown that prednisolone could reduce uNK cell concentrations, but there was no evidence of a significant beneficial effect on pregnancy outcomes (Cooper et al., 2019). The NNT per additional live birth was 6.55, and the results were quite different from traditional treatments. For example, the NNT of lymphocyte immunotherapy for the husband has been reported to be 10 (Porter et al., 2006). This suggests that the data could not be extended to all RSA patients, possibly because the inclusion criteria were more stringent than those in other studies or because the sample size of the present study was small.

Among clinical studies, in patients with thalassemia (Christiansen et al., 2005), aplastic anemia (Porter et al., 2006), and inflammatory bowel disease (Croh’s disease etc.) (Mahadevan et al., 2012; Coulam and Roussev, 2003) who are pregnant, long-term use of CsA has not been found to cause serious adverse reactions to the mother or fetus. Our previous research has also shown that CsA therapy is generally safe during pregnancy (Fu et al., 2016). In this study, there were no major adverse effects other than a congenital variation and mild local rash, which was consistent with a retrospective cohort study published in 2014 (Fu, 2015). Moreover, no differences were found in congenital malformations, Apgar scores or neonatal weights between the CsA group and the placebo group, indicating that there were no adverse effects of CsA intrauterine perfusion on the fetus. However, there was a lack of observation on the potential toxicity of CsA during pregnancy in this study. Animal experiments have shown embryo death and a reduced birth rate only in mice treated with doses 10,000 times that of humans (Groth et al., 2010). No adverse effects were found in rats (Renaud et al., 2011) or zebrafish (Shakhar et al., 2003). In conclusion, CsA is basically safe to use during pregnancy, but it should be carefully reserved for intractable alloimmune RSA.

Studies have shown that in the middle luteal phase, the number of endometrial NK cells in RSA patients is significantly higher than that in healthy women. However, Nathalie Ledde reported that the number and toxicity of NK cells in some repeated implantation failure (RIF) patients were decreased (Lédée et al., 2016). The increased number and toxicity of NK cells may promote excessive neovascular generation, induce an excessive inflammatory response and produce a high concentration of reactive oxygen species at the maternal-fetal interface, leading to rejection of the embryo by the mother, resulting in implantation failure or abortion. Nevertheless, the reduced number and toxicity of NK cells may not be conducive to new angiogenesis and the moderate inflammatory response required for the embryo implantation process, which also leads to implantation failure or abortion (Zhang et al., 2017). CD57 was expressed in NK cells that were highly mature, and NK cells with CD57^+^ were significantly increased in the decidua of 50% RSA patients (Vassiliadou and Bulmer, 1996; Luetke-Eversloh et al., 2014).

Recent studies have shown that peripheral blood and endometrial CD56^+^ cell and CD57^+^ cell concentrations of patients with RIF are significantly increased, and this increase is expected to become a new indicator for the evaluation of NK cells (Jiang et al., 2017). In this study, we used an advanced method to measure the concentration of uNK cells and found that intrauterine perfusion of CsA significantly reduced the concentration of uNK cells and improved pregnancy outcomes among patients with RSA. More importantly, the live birth ratio was not low in the placebo group, possibly because uNK cells were partially blocked by conventional treatment.

Taken together, our results provide strong evidence that intrauterine perfusion of CsA could be a promising treatment tactic for intractable alloimmune RSAs. Nevertheless, the efficacy and safety of CsA in all patients with immunological RSA is far from proven, and the number of pregnant women receiving CsA is insufficient to rule out any possible teratogenic effects. Therefore, intrauterine perfusion of CsA should be used with caution. To date, there have been no studies on the effects of intrauterine perfusion of CsA on the function of human reproduction, and the pathogenesis of the interaction between CsA, uNK cells and the immune system has not been clarified.

## Data Availability Statement

The raw data supporting the conclusions of this article will be made available by the authors, without undue reservation.

## Ethics Statement

The studies involving human participants were reviewed and approved by the Medical Ethics Committee of Qingdao Jinhua Hospital. The patients/participants provided their written informed consent to participate in this study.

## Author Contributions

LZ, LQ, and JF: conception and design, data analysis, and interpretation. LZ: administrative support. LQ, SB, YF, and LL: provision of study materials or patients, collection and assembly of data. All authors: manuscript writing and final approval of the manuscript.

## Conflict of Interest

The authors declare that the research was conducted in the absence of any commercial or financial relationships that could be construed as a potential conflict of interest.

## Publisher’s Note

All claims expressed in this article are solely those of the authors and do not necessarily represent those of their affiliated organizations, or those of the publisher, the editors and the reviewers. Any product that may be evaluated in this article, or claim that may be made by its manufacturer, is not guaranteed or endorsed by the publisher.
