# Corrigendum: Efficacy of Intrauterine Perfusion of Cyclosporin A for Intractable Recurrent Spontaneous Abortion Patients With Endometrial Alloimmune Disorders: A Randomized Controlled Trial

**DOI:** 10.3389/fphys.2021.774213

**Published:** 2021-10-12

**Authors:** Long Zhao, Lijuan Qi, Jinhua Fu, Shuqin Bi, Lin Li, Yinghui Fu

**Affiliations:** ^1^Department of Nephrology, The Affiliated Hospital of Qingdao University, Qingdao, China; ^2^Department of Obstetrics, Qingdao Jinhua Gynecology Hospital, Qingdao, China

**Keywords:** intrauterine perfusion of CsA, recurrent spontaneous abortion, uterine natural killer cells, CD56, CD57

In the published article, there was an error in affiliation 1, instead of “Department of Obstetrics, Qingdao Jinhua Hospital, Qingdao, China”, it should be “Department of Obstetrics, Qingdao Jinhua Gynecology Hospital, Qingdao, China”.

In the published article, there was an error in the affiliation order. The affiliation order should be

1 Department of Nephrology, The Affiliated Hospital of Qingdao University, Qingdao, China,

2 Department of Obstetrics, Qingdao Jinhua Gynecology Hospital, Qingdao, China

**Incorrect Author Order**

In the published article, there was an error in the order of authors. The author order should be

Long Zhao^1#*^, Lijuan Qi^2#^, Jinhua Fu^2^, Shuqin Bi^2^, Lin Li^1^ and Yinghui Fu^2^

#Long Zhao and Lijuan Qi contributed equally to this work.

The corrected Author Contributions Statement appears below.

LZ, LQ, and JF: conception and design, data analysis, and interpretation. LZ: administrative support. LQ, SB, YF, and LL: provision of study materials or patients, collection and assembly of data. All authors: manuscript writing and final approval of the manuscript.

The authors apologize for this error and state that this does not change the scientific conclusions of the article in any way. The original article has been updated.

## Publisher's Note

All claims expressed in this article are solely those of the authors and do not necessarily represent those of their affiliated organizations, or those of the publisher, the editors and the reviewers. Any product that may be evaluated in this article, or claim that may be made by its manufacturer, is not guaranteed or endorsed by the publisher.

